# Valorization of Olive Milling By-Products: Development and Application of an Antioxidant-Enriched Leavening Powder for Bakery Products

**DOI:** 10.3390/foods15091488

**Published:** 2026-04-24

**Authors:** Umile Gianfranco Spizzirri, Luigi Esposito, Donatella Restuccia, Pasquale Crupi, Donatello Fosco, Gianfranco Desideri, Domizia Vescovo, Maria Lisa Clodoveo, Maria Martuscelli, Francesca Aiello

**Affiliations:** 1 Ionian Department of Law, Economics and Environment, University of Bari Aldo Moro, 74123 Taranto, Italy; 2 Department of Bioscience and Technology for Food, Agriculture and Environment, University of Teramo, 64100 Teramo, Italy; 3 Department of Management, Sapienza University of Rome, Via del Castro Laurenziano 9, 00161 Rome, Italy; 4 Department of Agricultural, Food and Forestry Sciences, University of Palermo, Viale delle Scienze 13, 90128 Palermo, Italy; 5 Department for the Promotion of Human Science and Quality of Life, San Raffaele Roma University, 00166 Rome, Italy; 6 Interdisciplinary Department of Medicine, University of Bari Aldo Moro, 70125 Bari, Italy; 7 Department of Pharmacy, Health and Nutritional Sciences, University of Calabria, 87036 Rende, Italy

**Keywords:** olive pomace, bioactive compounds, antioxidant activity, leavening powder, functional food, shelf-life, gluten-free cookies

## Abstract

This research focuses on the synthesis of a novel baking powder enriched with bioactive molecules recovered from olive pomace via ultrasound-assisted extraction using a hydro-ethanolic mixture. The functional ingredient was engineered by anchoring the extracted phytocompounds onto a starch backbone through a sustainable grafting technique. Biscuits formulated with the innovative ingredient showed an increased concentration of phenolic compounds (2.162 mg GAE/g), encompassing both phenolic acids (0.372 mg GAE/g) and flavonoids (0.360 mg CTE/g). Enhanced antioxidant efficacy was recorded, mostly in aqueous media (IC_50_ = 0.554 mg mL^−1^ against ABTS radical) compared to organic environments (IC_50_ = 0.132 mg mL^−1^ against DPPH radical). Furthermore, Oxitest and oxidation stability reactor analyses revealed exceptional antioxidant capacity (induction period = 37 ± 2 h). By an accelerated shelf-life test, a marked instrumental color difference was observed with the fortified sample showing a darker, redder/brown color (ΔE > 16), as also confirmed by trained panelists. On the contrary, similar scores were achieved for the olfactory, textural and tasting attributes of the two samples, as well as values of the friability index (<1 mm^−1^) evaluated by instrumental techniques. This approach represents a sustainable strategy, transforming a high-polluting agri-food by-product into a source of bioactive compounds for nutritional and technological improvement of baked foods.

## 1. Introduction

Baked foods play a crucial role in the human diet. Among them, biscuits, or cookies, as they are commonly called in the US, are widely consumed in relation to their affordability, nutritional value, availability, ready-to-eat nature, and flavors. This worldwide diffusion is witnessed by the global market size that accounted for 16.38 billion USD in 2025 and is expected to reach 21.55 billion USD in 2031, with a compound annual growth rate (CAGR) of 4.68% in the next five years. Europe dominates the market, mostly driven by the Italian and Spanish demand, followed by North America [1].

With a moisture content of less than 5%, cookies are mainly composed of flour, sugar, fat, egg, milk, flavors, and leavening agents mixed in different proportions depending on the desired product [2]. Moreover, as consumers are more conscious about the effects of diet on wellbeing, new healthier solutions have been launched onto the market, including gluten-free (GF) and functional cookies [3].

As far as the former are concerned, it is well known that a GF diet is the only strategy to face celiac disease or any gluten-related disorder, despite great advancements in pharmaceutical treatments being achieved in recent years [4]. GF cookies are primarily aimed at consumers affected by celiac disease or gluten-related disorders, as well as individuals seeking “free-from” products [5]. Recent estimates indicate that celiac disease affects about 0.5–2% of the global population, with an average prevalence of around 1% and increasing diagnosis rates in Europe [6,7]. Consequently, the global gluten-free food market was valued at approximately 12.9 billion USD in 2024 and is expected to reach 33 billion USD by 2034, growing at a CAGR of around 9.9% [8]. Innovation in this segment focuses on improving texture, nutritional value, and shelf life, reflecting consumers’ increasing preference for healthier and tastier options.

Among GF baked products, cookies are a particularly suitable choice, as it has been demonstrated that the development of the gluten network is less important in cookies than in other GF leavened foods. This aspect represents a great advantage to overcome some of the technological and sensory drawbacks of other GF leavened foods [9]. In particular, short dough products with high fat and sugar were proven to possess suitable texture and spread, more in relation to starch gelatinization than to gluten network formation [10,11]. Great efforts were carried out to formulate functional biscuits as well. High protein, fiber-enriched and/or antioxidant-added applications were reported in the past decades [12,13,14,15,16].

Contemporary sustainable paradigms have increasingly focused on the valorization of agri-food by-products derived from fruit, vegetable, and seed processing as potent reservoirs of bioactive antioxidants [17]. These compounds are strategically utilized to augment both the nutritional profile and the oxidative stability of end-products [18,19,20,21,22]. Conventional fortification strategies typically involve the partial substitution of cereal flour with bioactive-rich flours, extracts, or powders. However, to the best of the authors’ knowledge, the literature remains void of instances where antioxidant properties are imparted to confectionery products through the functionalization of ingredients other than the bulk flour. In this context, the encapsulation or integration of antioxidant molecules within the leavening agent (baking powder) emerges as a compelling alternative. This approach yields a modular functional ingredient capable of being seamlessly integrated into a diverse array of baked goods formulations.

Baking powders are formed by leavening chemical agents (salts, acid compounds and starch) able to release gas when added to dough and producing, along with the water evaporation, the volume increase in the product [23]. Carbon dioxide is usually formed with or without ammonia, depending on the bicarbonate used [24]. Sodium bicarbonate is decomposed by heat to form sodium carbonate, CO_2_ and water, whereas ammonium bicarbonate produces ammonia, water, and CO_2_ at 59 °C. However, when used alone, sodium bicarbonate shows some drawbacks during heat decomposition, mostly related to the carbonate’s unpleasant taste, the undesirable crumb color, and the obtained CO_2_, whose amount is not the highest possible [25]. On the other hand, ammonium bicarbonate needs a low moisture environment to not leave any residue [26].

As sodium bicarbonate is the most used salt, to overcome the above-mentioned hindrances, acidic salts or complex salts acting as acids (several phosphates, tartaric acid, potassium bitartrate, glucono-d-lactone) are necessary in combination. Each acidic compound proved to have a characteristic and unique rate of reaction [27]. Depending on the different mechanisms of gas formation (decomposition or chemical neutralization), as well as on the speed at which the reaction takes place, the obtained product is strongly affected in terms of sensory and rheological features [28].

The last ingredient usually present in baking powders is starch, added to the mixture to avoid the premature reaction between the acidic and alkaline compounds and to absorb the moisture [29].

Olive pomace is the main solid by-product of olive oil extraction. Considerable amounts are produced, with literature reporting 35–40 to 70–80 kg per 100 kg of processed olives, and over 4.0 × 10^6^ m^3^ per year in EU countries [30,31]. Due to its high organic content and phenolic load, its management may pose an environmental challenge [32,33]. Olive pomace is primarily exploited for pomace oil production, but it has also found applications in animal feed, food fortification, energy generation, and the recovery of materials and bioactive compounds, such as phenolic compounds, lignin, and pectins [30,32,34,35,36,37,38,39]. The composition variability of olive pomace is mainly related to the genetic profile of the original drupe (i.e., cultivar), which can be typical of certain geographical areas [40]. Nevertheless, agronomic and technological parameters applied during olive oil production [35,41,42], also affect the relative abundance of the main oil pomace components (humidity, residual lipids, fiber, and phenolic compounds, among others).

In this context, a sustainable grafting procedure was adopted to anchor the antioxidant compounds extracted from olive oil pomace to the starch backbone, thus protecting the bioactive molecules during processing and avoiding unpleasant side effects [43]. The innovative leavening agent was used as an ingredient during the formulation of a GF cookie in order to address a potential consumer niche comprising both individuals affected by celiac disease or gluten-related disorders and health-conscious consumers seeking “free-from” functional products.

## 2. Materials and Methods

### 2.1. Reagents

All reagents and solvents were of analytical grade or higher unless otherwise specified and were purchased from commercial suppliers, including Phytolab (Aprilia, Italy), Merck (Darmstadt, Germany), Sigma-Aldrich (St. Louis, MO, USA), and VWR International (Milan, Italy). HPLC-grade water was obtained from Merck Life Science Srl (Milan, Italy). Ethanol and other solvents were purchased from VWR International (Milan, Italy). Folin–Ciocalteu reagent, sodium carbonate (Na_2_CO_3_), sodium hydroxide (NaOH), aluminum chloride (AlCl_3_), 2,2′-diphenyl-1-picrylhydrazyl radical (DPPH), and 2,2′-azino-bis(3-ethylbenzothiazolin-6-sulfonic acid) (ABTS) were purchased from Sigma-Aldrich (St. Louis, MO, USA).

### 2.2. Extraction Procedure of the Olive Pomace

In this study, olive pomace derived from the oil extraction process of two different olive cultivars, *Leccino* and *Carolea*, was utilized. These varieties were selected for their high industrial relevance. *Leccino* is one of the most widespread cultivars internationally, whereas *Carolea* represents a typical and economically significant variety of the Mediterranean basin, particularly in Southern Italy. The pomace from the *Leccino* cv was designated as LOPE, while the pomace from the *Carolea* cv was designated as COPE. Both LOPE and COPE samples were stored at −20 °C until processing. The extraction of bioactive compounds was carried out using two different solvent mixtures for each cultivar, to obtain extracts with varied polarity and selectivity, performing a preliminary screening of the compounds potentially present in the food by-product. For each extraction, 20 g of pomace sample was combined with 105 mL of organic solvent and 45 mL of purified water (70:30 *v*/*v*). The organic solvents employed in the extraction procedures were ethanol (1) and acetone (2).

The extractions were performed by an ultrasound-assisted extraction (UAE) using an Ultrasonic Bath ARGO^®^ at 40 °C, 40 KHz, 120 W (ARGO: Carpi, MO, Italy), for 30 min according to literature data, with some modifications [44]. After sonication, the solutions were centrifuged at 9000 rpm (relative centrifugal force, RCF = 9950× *g*) for 10 min to separate the solid residue from the supernatant. The supernatant was then subjected to rotary evaporation to remove the organic solvent under reduced pressure (water bath temperature of 37 °C and vacuum pressure of 100 mbar). Once the organic solvent was completely evaporated, the water residue was freeze-dried (lyophilization), yielding the final eight extracts, summarized in Table 1, in the form of stable, water-soluble powders, appropriately stored for further analysis.

### 2.3. Chemical Characterization of the Olive Pomace Extracts

The quali-quantitative characterization of the main polyphenols present in the freeze-dried pomace extracts, LOPE1 (4.0 mg), LOPE2 (9.8 mg), COPE1 (7.1 mg), COPE2 (7.0 mg), was carried out by an UHPLC 1290 Infinity (Agilent Technologies, Palo Alto, CA, USA) system, equipped with a quaternary pump, a column oven, an autosampler, and a diode array UV detector (DAD). The managed samples, solubilized with the same volume (~1.5 mL) of a solution 30:70 of water/organic solvent (i.e., ethanol or acetone), were injected (1.1 µL) onto an RP Zorbax Extend C_18_ 50 mm × 2.1 mm, 1.8 µm (Agilent Technologies, Palo Alto, CA, USA); mobile phase was a mixture of H_2_O/HCOOH 0.1% *v*/*v* (solvent A) and CH_3_CN (solvent B) and separation was performed by the following binary gradient: 0 min, 10% (B); 0.33 min 10% (B); 5.00 min 30% (B); 7.33 min 50% (B); 9.33 min 100% (B); 10.00 min 100% (B); 10.50 min 10% (B).

The column temperature was maintained at 40 °C, and the flow rate was 0.441 mL min^−1^. The chromatographic peaks were detected at 280, 320, and 360 nm wavelengths for determining simple phenols, phenolic acids, and flavonoids, respectively. Phenolic compounds were identified by matching different information, such as UV absorption maxima (λmax), UV spectra characteristics, and elution order compared to pre-injected analytical standards. Finally, identified compounds were quantified (Table 2) through calibration curves of 3-hydroxytyrosol (R^2^ = 0.9985) and luteolin (R^2^ = 0.9977) in the concentration range of 100–1.25 µg mL^−1^.

### 2.4. Synthesis of the Starch Conjugate

The synthesis of the polymer conjugate was performed from the extract COPE1, following the method reported in the literature [43], with some modifications. Briefly, in a reaction flask, 500 mg of corn starch was solubilized in 25 mL of purified water. Once the starch was fully dissolved, 12.5 mL of H_2_O_2_ (120 vol) and 250 mg of ascorbic acid were sequentially added. The solution was stirred after 2 h, and an appropriate amount of extract COPE1 (previously solubilized in 12.5 mL of purified water) corresponding to 105 mg of gallic acid, calculated via the Folin–Ciocalteu assay and expressed as mg of gallic acid/g extract, was added to the reaction mixture. The polymer solution was subsequently purified by dialysis (MWCO: 12–14,000 Da) in purified water for 72 h. After dialysis, the solution was frozen and lyophilized, yielding a highly soluble polymer powder. Additionally, a control polymer (blank polymer) was prepared under the same conditions but in the absence of the extract.

### 2.5. Preparation of Leavening Powder Containing the Conjugated Polymer

The antioxidant conjugate obtained from the grafting reaction was used as the main ingredient in the preparation of a functionalized leavening powder. The formulation of this leavening powder included 1.00 g of functionalized starch, 0.39 g of NaCl, 0.56 g of potassium tartrate, and 0.27 g of Na_2_CO_3_, resulting in a total of 2.22 g of leavening powder. All components were placed in a mortar and mixed using a pestle until a homogeneous powder was obtained. A control leavening powder (blank powder) was also prepared in the absence of the antioxidant polymer conjugate. Additionally, a commercially available leavening powder was used to test the antioxidant activity.

### 2.6. Preparation of Cookies

Three sets of cookies were prepared: B-CS, with commercial leavening powder; B-BS, with laboratory starch-based leavening powder; and B-SSE, with the studied functionalized leavening powder. Specifically, unsalted dairy butter was used as the lipid source to ensure a consistent textural profile and define the fat base for oxidative stability assessments.

A standard cookie recipe was followed [45], replacing conventional baking powder with the functionalized powder at an identical dosage to ensure comparability between the samples. The dough was shaped into disks (diameter 4 cm, thickness 0.5 cm) and baked in a preheated oven at 180 °C for 15 min. These conditions were chosen to achieve optimal leavening and sensory characteristics while monitoring the potential thermal degradation of the added bioactive compounds. The formulation, expressed in Table 3, reports both the specific amounts utilized for the laboratory-scale batch (yielding approximately five cookies) and the Baker’s percentage (where rice flour is assigned a value of 100%) to ensure process scalability and reproducibility. Although high temperatures can lead to a partial loss of thermosensitive phenolics, the functionalization of the starch carrier was designed to enhance the stability of the olive pomace extracts, preserving their antioxidant potential even after the baking process. The concentration of leavening powder (3.12%) was selected based on preliminary baking tests, which identified this ratio as the most effective for achieving a leavening performance and textural profile equivalent to the commercial control. All ingredients were purchased from a local supermarket.

### 2.7. Extraction Cookies Procedure

The three GF-cookie samples (B-SSE, B-CS and B-BS) were analyzed for their antioxidant activity on the same day of preparation, following an extraction method reported in the literature with some modifications [46]. For the extraction, 1 g of ground cookie was weighed and suspended in 7 mL of an ethanol/purified water (50:50, *v*/*v*) mixture. The mixture was then subjected to sonication for 15 min at room temperature. After sonication, the solution was centrifuged at 8000 rpm (RCF = 7871ּ× *g*) for 10 min, and the supernatant was collected and stored for subsequent analysis. Additionally, the same analyses were carried out over time (after 2, 4, and 6 days of storage at room temperature, 22 ± 2 °C).

### 2.8. Antioxidant Assays

#### 2.8.1. Total Phenolic Content Determination

The total phenolic content (TPC) of olive pomace extracts, starch conjugates, baking powder and cookie extracts was determined using the Folin–Ciocalteu assay, following a previously reported method with minor modifications [47]. Detailed experimental procedures are provided in Section S1 of the Supplementary Materials. The results were expressed as milligrams of gallic acid equivalent per gram of sample (mg GAE/g sample), based on a corresponding calibration curve.

#### 2.8.2. Total Phenolic Acid Determination

The total phenolic acid content (PAC) in olive pomace extracts, starch conjugates, baking powder and cookie extracts was determined using the Arnov test with minor modifications [48]. The protocol is described in Section S2 of the Supplementary Materials. The PAC was quantified as milligrams of gallic acid equivalent per gram of sample (mg GAE/g sample), based on a corresponding calibration curve.

#### 2.8.3. Flavonoid Content Determination

The flavonoid content (FC) of the olive pomace extracts, starch conjugates, baking powder and cookie extracts was assessed using a method adapted from the literature [49]. Specific details are reported in Section S3 of the Supplementary Materials. Flavonoid levels were quantified as milligrams of catechin equivalent per gram of sample (mg CTE/g sample), using an appropriate calibration curve.

#### 2.8.4. Scavenger Activity Against DPPH Radical

The antioxidant activity of the olive pomace extracts, starch conjugates, baking powder and cookie extracts in an organic environment was measured by evaluating their ability to reduce the 2,2′-diphenyl-1-picrylhydrazyl (DPPH) radical. The assay was conducted based on a literature method with slight modifications [50] (see Section S4 in the Supplementary Materials section). The scavenging activity against the lipophilic DPPH radical was reported as the Antiradical Power (ARP = 1/IC_50_), where IC_50_ represents the concentration (mg mL^−1^) required to inhibit 50% of the initial radical species.

#### 2.8.5. Scavenger Activity Against ABTS Radical

The antioxidant activities of the olive pomace extracts, starch conjugates, baking powder and cookie extracts were evaluated by assessing their ability to reduce the radical species 2,2′-azino-bis (3-ethylbenzothiazoline-6-sulfonic acid) (ABTS). The method was adapted from a previously reported procedure with minor modifications [51] (see Section S5 in the Supplementary Materials section). The scavenging activity of the tested samples was expressed as Antiradical Power (ARP = 1/IC_50_), calculated from the IC_50_ values (mg mL^−1^).

### 2.9. Technological Properties Evaluation of Cookies

#### 2.9.1. Oxidative Stability

The Oxidative stability was assessed by means of OXITEST (VELP, Usmate, MB, Italy), carried out on cookies (10 g of grounded product). Accelerated oxidation test was carried out under over-pressure of pure oxygen (0.6 MPa, degree 5.0) and constant high temperature (100 °C) [52]. The OXITEST response is the induction period (IP), expressed as “stability time” before fat oxidation and corresponding to a drop of O_2_ pressure due to the consumption of oxygen by the sample. The IP value was automatically calculated from the oxidation curve by graphical method (two tangent methods) by using the OXISoftTM program included in the instrument. With this procedure, it has been possible to extrapolate and estimate the oxidation stability of samples at room temperature, in case of linear dependence on the temperature.

#### 2.9.2. Sensory Test

Volunteers from the food science Department of the University of Teramo (UNITE) were recruited and trained to perform a panel test. Sensory analyses followed UNITE’s ethics and data protection protocols. Participants gave informed consent before participating in the analysis. They were informed that they were able to withdraw at any time without giving a reason, they were provided with allergen information and with the following information about personal data processing: “all data captured will be confidential and anonymized and will be only used for research purposes”. The tested products were safe for consumption.

Cookies obtained using the functional and the commercial baking powders (B-SSE and B-CS, respectively) underwent a sensory test through a Qualitative Descriptive Analysis (QDA). Judges were screened for their ability to discriminate odors and tastes and then trained for vocabulary development and consensus about sample testing. Multiple training sessions were done until reaching a shared consensus about intensity and vocabulary development. Ten trained panelists took part in the sensory test; five women and five men with an average age of 35.8 years old. Round-shaped cookies of about 4.5 g were simultaneously served on white dishes with random numeration. The panel test was performed in the sensory science laboratory of the University of Teramo, respecting guidelines from ISO 8589:2007 [53]. All samples were evaluated for six classes of descriptors grouped as: appearance, odor, off odor, taste, flavor, and texture. Instructions to participants were repeated; information about descriptors was given, and the scale of intensity was explained. Panelists were asked to assign a score for each descriptor from 1 to 9, where 1 represented the absence of the attribute and 9 the maximum intensity; individual templates for each sample were used. Between sample tasting, participants drank still water to rinse their mouths. The evaluation was performed in individual cabinets under white D65 light. To better define classes of descriptors, a review of the recent literature about bakery/extruded and snack products was also done, considering the exclusion of gluten-forming ingredients in the formulation [54,55,56].

#### 2.9.3. Accelerated Shelf Life (ASL) Test

A test of accelerated shelf life was also carried out on cookie samples packaged according to a commercial procedure. After complete cooling at room temperature, 5 cookies for each trial were packaged in co-extruded polypropylene 25 µm coupled with polypropylene cast 25 µm film (Poplast srl, Castel Sangiovanni, Piacenza, Italy); bags were hermetically sealed (MOTOKRIMPER, NAIS, Milan, Italy).

Packaged samples were stored at 40 °C and controlled R.H. (55%) with no light for 42 days in an Incubator model (FOC 225 E, Velp Scientifica, Usmate Velate, MB, Italy). The sampling for the analyses of moisture content, water activity (a_w_) and physical characteristics was performed immediately after preparation (0 days, t_0_) and after 7 days (t_7_), 14 days (t_14_), 21 days (t_21_), and at the end of the period of observation (t_42_). The trial was replicated on two independent occasions.

Moisture content was measured according to the gravimetric AOAC 925.09 method [57] using an oven method at 105 °C for 12 h.

The values of water activity (a_w_) were obtained with the Aqualab 4 TE kit (Court Pullman, WA, USA).

Mechanical properties were evaluated, modifying the method reported by Martuscelli et al., [58], through a dynamometer Instron mod. 5542-H5036 (Instron International Limited, High Wycombe, UK) equipped with a maximum load of 500 N. Round-shaped cookies of about 4.5 g, 0.5 cm in thickness, were used as specimens for a shear test. The analysis (at room temperature, 22 ± 2 °C) was carried out using a ten-bladed Kramer head at the maximum velocity of 80 mm min^−1^. The specimens were placed in the middle of the cell, allowing the head to find the most uniform surface. Maximum force (F_max_) expressed in N was used as the index of the maximum hardness [59]; total energy (E_t_) expressed in J was used as an index of the whole energy amount required to cut and extrude the product [60]; the reciprocal of the E_t_/F_max_ ratio, index of the degree of deformability, was used as the friability index (mm^−1^) [58].

Color was determined by a colorimeter CR-5 (Spectrally based, Konica Minolta, Tokyo, Japan) with D65 light source and observer 10°. The analysis was carried out in different locations of cookie samples (at least nine specimens, for each formulation). Color was expressed as L* (lightness, intensity of white color), a* (+a, red; −a, green) and b* (+b, yellow; −b, blue) values. The coordinates a* and b* were used to calculate hue angle value [= arctan (b*/a*)], and chroma or saturation index [= (a*^2^ + b*^2^) 1/2]. The color difference (ΔE) between the sample color (L_2_*, a_2_*, b_2_*) and the reference color (L_1_*, a_1_*, b_1_*) was determined according to the following Equation (1) [61]:

(1) $$\Delta E=\sqrt{{\left(L_{1}^{*}-L_{2}^{*}\right)}^{2}+{\left(a_{1}^{*}-a_{2}^{*}\right)}^{2}+{\left(b_{1}^{*}-b_{2}^{*}\right)}^{2}}$$

### 2.10. Statistical Analysis

All experiments were carried out in triplicate. Data were expressed as mean ± standard deviation (S.D.). Significance was determined using a one-way analysis of variance (ANOVA), followed by Duncan’s multiple range test at a significance level of *p* < 0.05. The concentration required to achieve 50% inhibition (IC_50_) was calculated by nonlinear regression using GraphPad Prism version 4.0 for Windows (GraphPad Software, San Diego, CA, USA). The dose–response curve was obtained by plotting the percentage of inhibition against concentration.

## 3. Results and Discussion

### 3.1. Extraction of Olive Pomace Extracts

The recovery of bioactive constituents from solid residues generated during olive milling was effectively achieved via ultrasound-assisted extraction (UAE). This process utilized binary mixtures (7:3 *v*/*v*) of water and miscible organic solvents, specifically ethanol and acetone. Acetone was employed as a technical benchmark due to its high efficiency in disrupting hydrogen bonds and polyphenol-protein complexes, which often makes it a superior solvent for the recovery of complex phenolics from lignocellulosic matrices. By comparing its performance with ethanol, it has been possible to evaluate whether a more sustainable solvent system could provide comparable extraction yields. Current literature suggests that hydro-organic systems circumvent the inherent limitations associated with purely lipophilic solvents such as chloroform, hexane, or ethyl acetate [62]. This comparative framework facilitated the identification of an optimal and sustainable solvent system (hydro-ethanolic mixture) able to efficiently partition the primary classes of bioactive compounds. The superior performance of binary-solvent systems over single-solvent choices in the extraction of phenolics from vegetable matrices is well-documented [63]. The incorporation of an aqueous fraction into an organic solvent modulates the medium’s polarity, thereby enhancing the solvating power for polyphenolic species [47]. This phenomenon is governed by the interplay of the solvent polarity and selectivity, which dictates the solubilization of compounds based on their specific physicochemical attributes. Solvents with intermediate dielectric constants, such as hydro-alcoholic mixtures, favor the extraction of phenolics through the establishment of hydrogen bonding and polar interactions with hydroxyl moieties [64,65]. Consequently, the fine-tuning of the water-to-organic ratio enables precise control of selectivity, significantly augmenting the yield of target bioactives.

On the other hand, the UAE allows extraction at low temperatures with short processing times, reducing solvent consumption, preventing thermal damage, and preserving structural and bioactive properties [66]. UAE represents a cost-effective alternative to other eco-friendly extraction technologies, maintaining high efficiency while reducing operational expenses [39]. Comparative studies evaluating UAE, microwave-assisted extraction, and pressurized liquid extraction for polyphenol recovery from olive pomace have highlighted their advantages [67]. Additionally, the UAE has been successfully applied using water as a solvent for polyphenol extraction from olive pomace [68]. The experimental conditions were optimized through a detailed analysis of key parameters such as ultrasonic power, extraction time, and the sample-to-solvent ratio, identifying optimal conditions at 0.133 g mL^−1^ for 30 min at 40 °C. These conditions enhanced the extraction efficiency, producing polyphenol-rich extracts with strong antioxidant properties [63,68].

The extraction conditions employed, along with yields and extraction efficiencies, expressed in grams of dry sample obtained after complete evaporation under reduced pressure, are reported in Table 1. Regarding extracts obtained from the *Leccino* cultivar, extractions conducted with water/ethanol and water/acetone mixtures produced similar yields in terms of both extraction efficiency and the amount of dry powder obtained. A similar trend was observed in extracts obtained from the *Carolea* cultivar, although the quantities and yields of each extraction were higher compared to those obtained from the *Leccino* cultivar. This variation highlights how the genetic background of the cultivar influences the quantitative recovery of bioactives, with *Carolea* showing a higher predisposition for polyphenol release under the same UAE conditions. Overall, these findings support the effectiveness of the solvent screening approach and confirm that the hydroalcoholic system provides consistent performance across different cultivars while maintaining higher environmental sustainability.

### 3.2. Characterization of the Olive Pomace Extracts

#### 3.2.1. Antioxidant Performance of Olive Pomace Extracts

The total phenol content (TPC) was evaluated using the Folin–Ciocalteu assay, while phenolic acid (PAC) and flavonoid content (FC) were quantified via specific colorimetric assays (Table 4). The results observed were consistent across all tests for both analyzed cultivars. Furthermore, a partial correlation can be observed between the TPC values of the extracts and their antioxidant performance, which is closely linked to the hydrophilic/lipophilic balance of polyphenolic molecules in each extract [51]. It should also be noted that the presence of other non-phenolic compounds in the extracts, such as sterols [69] and terpenes [70], may significantly contribute to the antioxidant properties of the samples.

The ability of the extracts to inhibit the DPPH radical, expressed as Antiradical Power (mL mg^−1^), is reported in Table 4. Analysis of the corresponding dose–response curves reveals that the extracts obtained using the ethanol/water mixture from the *Carolea* and *Leccino* cultivars exhibit higher ARP values (9.901 and 12.195 mL mg^−1^, respectively) compared to the other extracts. These findings are consistent in magnitude with the values reported by Cioffi et al. [71], who investigated the antioxidant properties of olive milling pomace extracts from various regions of the Cilento area in Southern Italy.

A detailed analysis of the inhibition profiles of the extracts against the ABTS radical confirms that they exhibit good performance even in an aqueous environment. According to Floegel et al. [72], the ABTS radical scavenging assay provides a more accurate estimation of the antioxidant capacity of food matrices, including fruits, vegetables, and beverages. Furthermore, Prior et al. [73] reported that smaller antioxidant compounds with lower steric hindrance interact more efficiently with the unpaired electron of the DPPH radical, potentially leading to an overestimation of their actual antioxidant capacity and, consequently, to misleading results. Therefore, Floegel et al. [72] suggest that the ABTS radical inhibition assay is a more reliable method for assessing the antioxidant activity of food matrices, as it accurately detects the response generated by hydrophilic compounds. Among the tested extracts, COPE1 exhibited the highest antioxidant activity (ARP = 32.258 mL mg^−1^). This result is consistent with the high TPC value recorded for COPE1 and the lower toxicity of ethanol compared to other organic solvents, supporting its selection as the most suitable extract for the development of polymeric derivatives.

#### 3.2.2. Phenolic Profile in Olive Pomace Extracts by UHPC-DAD Analysis

Table 2 itemized the phenolic compounds identified by matching their chromatographic and spectrophotometric features with those of the available pure standards. The presence of these compounds in olive pomace was previously reported in the literature, with values of a similar order of magnitude [74]. They were selected in the profiling analysis based on their superior antioxidant activity (attributed to structural features such as multiple hydroxyl groups, catechol moieties, conjugated double bonds, and a 4-oxo function) coupled with favorable bio-absorption properties, as evidenced by pharmacokinetic studies demonstrating rapid absorption of tyrosol and hydroxytyrosol, and their distinct solubility profiles, which differ between the more polar simple phenols (tyrosol and hydroxytyrosol) and flavonoids (luteolin and apigenin) [75,76].

As evident from statistical analysis, olive pomace extracts in water/ethanol, both of *Leccino* and *Carolea* (LOPE1 and COPE1, respectively), showed the highest content of 3-hydroxytyrosol and tyrosol. As simple phenols have an elevated antioxidant capacity, these data seem in agreement with the calculated values of the extracts (Table 4), considering that DPPH radical, in particular, prefers to interact with less sterically bulky and low molecular weight antioxidant compounds [73]. Regarding the flavonoids, the water/ethanol mixture appeared to be the best extraction solvent just in the case of *Leccino* pomace; indeed, in LOPE extracts, either luteolin or apigenin was quantified at 5.8 µg g^−1^, an amount of almost threefold greater than the other extracts (Table 2). Conversely, the same flavonoids were better extracted from *Carolea* pomace by water/acetone (COPE2).

### 3.3. Synthesis and Characterization of the Olive Pomace Extract and Starch Conjugate

The selection of corn starch as a carrier was based on its dual functionality: its essential technological role in ensuring the stability and processability of leavening agents, and its chemical architecture, which is replete with hydroxyl moieties conducive to the grafting mechanism. The functionalization of the starch backbone with bioactive constituents from the COPE1 extract was executed via radical grafting, resulting in a macromolecular conjugate endowed with robust antioxidant properties for multifaceted applications. Such polymeric conjugates constitute advanced systems that offer superior chemical stability and attenuated degradation rates compared to their low-molecular-weight counterparts [77], thereby expanding their utility across the biomedical, cosmetic, and food sectors. Whereas traditional polymerization protocols frequently necessitate arduous purification and stringent reaction conditions, this study employed a facile, eco-friendly grafting strategy mediated by a hydrogen peroxide/ascorbic acid redox-initiator system [43]. This approach facilitates polymerization under ambient conditions, thereby mitigating the thermal degradation of heat-sensitive compounds and preventing the formation of deleterious intermediates [78].

The process included two stages: the activation of the starch glucidic chains to form the radical sites, followed by the grafting of the antioxidant molecules [79]. The optimal COPE1-to-starch ratio was 210 mg gallic acid equivalent per gram of starch, determined via the Folin–Ciocalteu assay. After the synthesis, the polymeric conjugate (labeled SSE) was dialyzed to remove unreacted compounds and lyophilized to obtain a highly soluble polymeric powder. A control polymer (labeled BS) was also synthesized under the same conditions but without the extract.

To evaluate the antioxidant properties of the polymeric derivative, the same assays were performed as described previously. The data in Table 5 present the TPC, PAC, and FC values for SSE, BS, and commercial starch (CS), as well as their antioxidant activity against various radical species. The results demonstrate the success of the grafting reaction, as BS showed no positive results in any assay. It exhibited a negative outcome in the TPC value and did not respond in assays for phenolic acids, flavonoids, or antioxidant capacity against DPPH and ABTS radicals, indicating that the antioxidant activity is solely attributed to the active components in COPE1. The inhibition profiles reveal that SSE shows good radical inhibition in both organic and aqueous environments, with a more pronounced response against the ABTS radical (IC_50_ = 0.180 mg mL^−1^), which is six times higher than the corresponding extract.

### 3.4. Preparation and Characterization of the Leavening Powder Based on Functionalized Starch

The polymeric conjugate was incorporated into a leavening powder formulation for baked goods. The leavening powder was prepared as detailed in Section 2.5. Baking powder, which is both flavorless and odorless, is employed in both sweet and savory applications to facilitate the rising of dough, resulting in a lighter and fluffier texture. It contributes to the production of easily digestible, soft, and light-textured baked goods. Additionally, baking powder is suitable for vegan and vegetarian diets, as it is free from animal-derived ingredients and composed entirely of natural substances. The primary ingredients include sodium bicarbonate and potassium tartrate, which serve as leavening agents, enhancing the expansion and increasing the volume and tenderness of baked products [80]. Moreover, the leavening powder can be useful in the preparation of a variety of baked goods, imparting notable antioxidant properties to each product while preserving its organoleptic qualities and offering potential health benefits. As shown in Table 5, the functionalized leavening powder (LP-SSE) yields positive results in various antioxidant assays. In contrast, both leavening powders based on BS (LP-BS) and CS (LP-CS) return negative results in all assays. Therefore, the observed antioxidant activity can be attributed exclusively to the biopolymer present as the main component in the functionalized leavening powder.

### 3.5. Antioxidant Properties of Functional Cookies

The quantification of phenolic compounds and the evaluation of their antioxidant activity were essential to determine whether the innovative product under investigation could offer potential health benefits. The data in Table 6 show that the functionalized cookie (B-SSE) contains significantly higher amounts of polyphenols compared to B-CS and B-BS. These displayed a slight positive result in the Folin–Ciocalteu test, likely due to the presence of certain ingredients used in the preparation of the cookies that may contribute to this outcome. The TPC of the cookies (t = 0 day) was quantified in 2.16 mg GAEg^−1^, while PAC and FC values were 0.372 and 0.360 mg CTE g^−1^, respectively. These data are in the same order of magnitude as cookies supplemented with plant extracts, ranging from 26.44 to 106.7 mg GA 100 g^−1^ for cookies enriched with *Lonicera japonica* Thunb (4% *w*/*w*) [39], and cookies with a 5% level of camu-camu coproduct powder having a TPC of 0.12 mg GA g^−1^ [81].

A careful analysis of the inhibition profile against radical species shows that the B-SSE exhibits superior antioxidant performance against the hydrophilic ABTS radical (ARP = 0.926 mL mg^−1^) compared to the lipophilic DPPH radical (IC_50_ = 0.132 mg mL^−1^), supporting the previous results recorded for both the leavening powder and the polymeric derivative. Data collected in Table 6 further confirm that B-BS and B-CS gave negative results in the antioxidant assays.

In order to evaluate antioxidant features over time, at predetermined time intervals, each cookie was subjected to the usual colorimetric tests. Table 6 reports the data regarding the quantity of polyphenols, phenolic acids, flavonoids, and antioxidant activity of the cookies against ABTS and DPPH radicals at time zero (t = 0 days), after two days (t = 2 days), after four days (t = 4 days), and after six days (t = 6 days).

The analysis of IC_50_ values over time highlighted the superior radical-scavenging activity of the food matrix prepared with B-SSE in aqueous conditions. Specifically, lower concentrations of the cookie were needed to achieve a 50% reduction in the initial ABTS radical concentration (0.478–0.554 mL mg^−1^). In organic conditions, higher concentrations of functionalized cookies were required to produce a 50% decrease in the initial DPPH radical concentration (0.122–0.132 mg mL^−1^). Temporal analysis of the various parameters demonstrated that B-SSE maintained its properties almost unchanged, as evidenced by only a minimal increase in the ARP values for ABTS and DPPH radicals after 6 days (3.472 and 1.555 mL mg^−1^, respectively). Based on these results, it can be concluded that cookies formulated with the functional leavening agent made from a starch-olive pomace extract conjugate possessed improved antioxidant features compared to conventional products.

### 3.6. Technological Properties of Cookies

**Figure 1:**
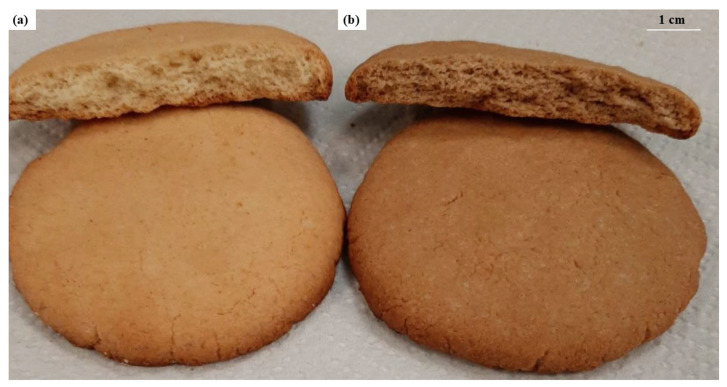
Representative samples of cookies obtained using: (**a**) the commercial leavening agent (B-CS, control); (**b**) the new leavening agent (B-SSE, functionalized cookie).

To evaluate the real-world applicability of the newly developed ingredient, the technological and sensory evaluations focused on a comparison between the functionalized cookie (B-SSE) and the commercial benchmark (B-CS). This approach was chosen to demonstrate the potential of the innovative leavening powder to replace traditional commercial additives. The starch-based control (B-BS), having served its primary purpose of confirming that the antioxidant activity was solely due to the grafted phytocompounds (as seen in the preliminary screening), was excluded from the oxidative stability and sensory profiling to streamline the comparison against the industrial standard. Figure 1 illustrates a comparison of the thickness of the functionalized cookie and the commercial cookie. The leavening of the functionalized cookie is evident from the visible pore size in the cookie section, which is similar to that of the commercial cookie.

Products like cookies, crackers, and breadsticks that are rich in fat and low in moisture are more susceptible to lipid oxidation. For these products, lipid oxidation is considered the main cause of deterioration [52]. In this study, the OXITEST reactor was used to monitor the oxidative stability of B-CS and B-SSE. The OXITEST response is the induction period (IP), expressed as “stability time” before the oxidation starts. This test allowed for the quick evaluation of the oxidative stability of samples in the presence of pressurized oxygen at relatively high temperatures. Results are reported in Table 7 (and Figure S1). Data showed that the products have different stabilities in the oxidation reaction. In the case of B-SSE, the IP is significantly (*p* < 0.05) higher compared to that of the control sample (B-CS). This different oxidation stability could be related to the positive effect of olive pomace extracts present in sample B-SSE. Despite being used at low concentration, the fortified baking powder has a strong potential for reducing oxidative phenomena in baked goods. In this sense, it could be a more convenient solution for consumers who prefer foods “free” from synthetic additives (e.g., butylated hydroxyl anisole), often associated with a possible toxicity [82].

Moreover, it was observed that natural antioxidants from plant extracts improved antioxidant properties in biscuits, in comparison with BHA [83].

**Figure 2:**
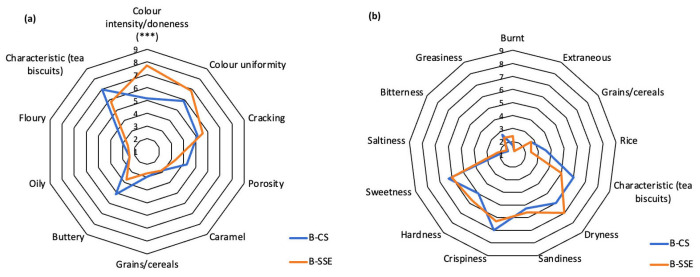
Descriptive sensory analyses of the experimental sample (B-SSE, orange line) and control (B-CS, blue line): (**a**) visual, tactile and flavor descriptors; (**b**) taste and texture descriptors. Asterisks indicate significance at *** *p* < 0.001.

The qualitative descriptive analysis (QDA), performed immediately after preparation (t_0_), confirmed that color is the only descriptor significantly affected by the addition of the innovative bakery powder (Figure 2a,b); meanwhile, similar scores were achieved by the two samples examined for the olfactory, tasting, textural and other attributes.

Accelerated shelf-life test demonstrates a high stability of qualitative characteristics in newly formulated cookies, packaged and stored in stressed conditions (40 °C, R.H. 55%). In this stage, the B-SSE sample was compared with the commercial control (B-CS) to verify if the experimental leavening powder could maintain technological standards equivalent to those of a commercial reference during storage. Packaged samples B-CS and B-SSE had moisture contents and water activity values (see Table 8) similar to those found in different studies concerning gluten-free biscuits made with rice flour [84,85]. Moisture content affects the sensory attributes and physical properties of bakery products [86]. Anyway, values lower than 4% limit the occurrence of remarkable changes [87]. Additionally, a_w_ values lower than 0.5 make products safe from microbial spoilage [88]. A significant (*p* < 0.05) decrease in moisture occurred both in the control (B-CS) and the experimental product (B-SSE), at the final stages of storage in stressed conditions.

Several studies have seen that rice flour has lower moisture content than wheat flour, resulting in drier final products [89]. In a recent study, Banu Itagi et al. [85] have tested the effect on cookies of different flours (whole grain rice and wheat) and sugars. Authors demonstrated that white sugar has a greater influence on moisture reduction in respect of brown sugar and jaggery. Higher inverted sugars and mineral ions values were found responsible for higher moisture content and a_W_; so, flour provenience has not been detrimental for moisture content and a_W_ as sugars. In line with these considerations, only white sucrose was employed for the samples, which may explain these reduced levels. Further information was provided by Pestorić et al. [90] who found that higher temperatures (40 °C vs. 23 °C) reduced the a_W_ values of gluten-free biscuits independently of the packaging presence.

Results of texture analysis are also reported in Table 8. The olive pomace extracts did not significantly (*p* > 0.05) affect the deformability and friability of cookies, unlike when olive pomace is directly added as an ingredient in the formulation [91]. It can be assumed that the extraction protocol proposed in this study does not allow the retention of other compounds, such as fibers and proteins, responsible for hardening the biscuit dough, as reported by Banu Itagi et al. [85]. This trend was also recorded for other GF foods as reported by Esposito et al. [54]. Evaluations on a bakery product added with carob flour proved that fibers, proteins, and long-chain sugars influenced moisture content, a_W_, hardness, and color [54].

Conversely, the addition of the functionalized powder significantly influenced (*p* < 0.05) colorimetric characteristics (Table 9), leading to a decrease in lightness (L*), an increase in redness (a*) and a decrease in yellowness (b*) parameters in experimental cookies, with respect to the control. A similar trend was observed by other authors in fortified pasta with olive pomace [92], in wheat breadsticks, added 5–10% olive pomace [93], and in breadsticks enriched with olive mill waste waters extracts, where authors also noticed a reduced hardness and a higher moisture, due to the higher water content of the extracts [94].

As shown in Table 10, no color difference resulted (ΔE < 3), demonstrating high color stability for both of the sample along time of storage. Furthermore, as expected, ΔE was very high (>6) at each observation time, comparing B-SSE with B-CS (as reference sample).

## 4. Conclusions and Future Perspectives

This study demonstrated the feasibility of valorizing olive pomace, a by-product of olive oil production, as a source of functional bioactive compounds for application in bakery products. The results provided solid proof-of-concept for the development of a functional leavening powder through an eco-friendly grafting procedure. In this context, a preliminary screening using water-miscible solvents with different polarities was carried out to assess extraction selectivity and efficiency, allowing the identification of the most sustainable system capable of recovering the main classes of bioactive compounds, particularly phenolics. These compounds were successfully grafted onto starch via an eco-friendly procedure, producing a functionalized leavening powder. When applied to gluten-free cookie production, this ingredient enhanced the antioxidant properties of the final product while maintaining its organoleptic and technological quality. The approach proposed in this research is supported by robust analytical and sensory data and thus represents a valuable contribution to the development of clean-label and environmentally responsible functional foods, while promoting the circular economy through the valorization of agro-industrial waste.

Although promising, some limitations of the study cannot be neglected, especially in the case of large-scale applications. Indeed, the research claims are currently grounded at a laboratory scale. First of all, to obtain proper quantities of extract, great amounts of waste should be handled and freeze-dried as the extraction yield is quite low. It follows that high levels of solvents should be applied, impacting costs, mostly in relation to ethanol. Second, even if the functionalization of the polymer requires mild conditions and non-toxic reactants, the procedure is not immediate and requires specific technical skills. Moreover, considering the ratio extract/polymer in the conjugate, to prepare the functional ingredient at the industrial level, high extract concentrations and thus longer times would be necessary. In this sense, prototypes should be necessary to evaluate yields and reaction characteristics that at the large-scale level could be different in comparison to the laboratory level. Current findings should therefore be considered as a starting point for further scale-up studies. Third, the olive oil pomace is available during the olive oil production, which is usually concentrated in time (October–December) and space (Mediterranean basin). This implies that the raw materials should be bought all at once and stored for most of the year. This aspect would require investments, technologies and devoted areas for the purpose by the producing factories.

Nevertheless, some unquestionable advantages deserve to be underlined. First, a noble use for highly polluting agri-food waste can be offered, reducing disposal costs and promoting environmental sustainability and the circular economy at the same time. Second, a valuable food ingredient can be readily obtained. As it avoids toxic chemical substances during preparation, it can be considered safe for human consumption and does not alter the baking powder formulation, as the starch is already present in the list of ingredients. Third, the new baking agent could be widely applied to any baked food thanks to its flexibility. In this regard, the research put in evidence that with only one procedure, it was possible to obtain one innovative ingredient and possibly many functionally derived foods. Fourth, the final product combined an improved shelf-life with health-promoting traits with almost no effects on organoleptic features. Stability data from Oxitest and accelerated shelf-life tests further strengthen the claim that this ingredient can effectively replace synthetic additives. These aspects are also more relevant, considering that the biscuits were designed for celiac consumers whose disorders reached, at the global level, a prevalence of up to 1%.

Looking ahead, future research should aim at bridging the gap between laboratory findings and real-world applications. Moving from benchtop results to industrial prototypes is essential. Prototype-scale trials are essential to validate the process efficiency, stability, and cost-effectiveness at an industrial level. Furthermore, while this study demonstrates the superior performance of the functionalized powder compared to a standard commercial leavening agent, future research could benefit from a direct comparison with synthetic antioxidants commonly used in the bakery industry. Such a comparison would help to fully quantify the effectiveness of these bio-based alternatives against traditional chemical stabilizers, providing a more comprehensive overview of their potential for large-scale industrial adoption in the context of clean-label food production. Furthermore, in vivo studies are necessary to confirm the physiological benefits of antioxidant-enriched cookies, especially regarding glycemic index modulation and antioxidant bioactivity in humans. Consumer acceptance testing should also be performed to evaluate the sensory perception and purchase intention associated with these new-generation cookies. Finally, techno-economic and life cycle assessments will be required to define the competitiveness, scalability, and overall sustainability of this valorization strategy within the functional food industry.

## Figures and Tables

**Table 1:** Ultrasound-assisted extraction of *Leccino* and *Carolea* olive pomace. Results are expressed as mean ± S.D. (*n* = 3), *p* < 0.05.

Samples		Extraction Conditions			Yield		
Code	Matrix (g)	Extraction Solvent	V (mL)	T (°C)	t (min)	Mass (g)	%
LOPE1	20.0	Water/Ethanol 30/70 (*v*/*v*)	150	40	30	0.75 ± 0.03 ^a^	3.75 ± 0.15 ^a^
LOPE2	20.0	Water/Acetone 30/70 (*v*/*v*)	150	40	30	0.78 ± 0.03 ^a^	3.89 ± 0.16 ^a^
COPE1	20.0	Water/Ethanol 30/70 (*v*/*v*)	150	40	30	1.18 ± 0.05 ^b^	5.80 ± 0.20 ^b^
COPE2	20.0	Water/Acetone 30/70 (*v*/*v*)	150	40	30	1.23 ± 0.05 ^b^	6.12 ± 0.21 ^b^

LOPE: *Leccino* cv Olive Pomace Extract; COPE: *Carolea* cv Olive Pomace Extract; (1) water/ethanol; (2) water/acetone. Different letters are significantly different at *p* < 0.05.

**Table 2:** UHPLC-DAD identification and quantification of polyphenols profile in *Leccino* and *Carolea* olive pomace extracts.

Code	3-Hydroxytyrosol (μg/g)	Tyrosol (μg/g)	Luteolin (μg/g)	Apigenin (μg/g)
LOPE1	1.02 ± 0.11 ^a^	73 ± 7 ^a^	5.8 ± 0.6 ^a^	5.8 ± 0.5 ^a^
LOPE2	n.d.	2.8 ± 0.2 ^d^	2.02 ± 0.18 ^b^	1.98 ± 0.16 ^b^
COPE1	0.41 ± 0.03 ^b^	44 ± 5 ^b^	0.68 ± 0.09 ^d^	0.89 ± 0.10 ^c^
COPE2	n.d.	2.1 ± 0.2 ^c^	1.55 ± 0.16 ^b,c^	1.91 ± 0.14 ^b^

LOPE: *Leccino* cv Olive Pomace Extract; COPE: *Carolea* cv Olive Pomace Extract; (1) water/ethanol; (2) water/acetone. Different letters are significantly different at *p* < 0.05. n.d.: not detected.

**Table 3:** Formulation of functionalized biscuits.

Ingredient	Amount (g)	Baker’s Percentage (%)
Rice flour	60.0	100.00
Sugar	27.0	45.00
Butter	14.0	23.33
Egg	12.0	20.00
Water	6.0	10.00
Functionalized leavening powder	1.87	3.12
Total	120.87	201.45

**Table 4:** Antioxidant properties of *Leccino* and *Carolea* olive pomace extracts. Results are expressed as mean ± S.D. (*n* = 3), *p* < 0.05.

Code	TPC (mg GAE/g)	PAC (mg GAE/g)	FC (mg CTE/g)	ARP (mL mg^−1^)	
				DPPH Radical	ABTS Radical
LOPE1	48.33 ± 2.10 ^b^	42.90 ± 1.85 ^a^	33.77 ± 1.47 ^b^	12.195 ± 0.445 ^a^	23.809 ± 0.566 ^b^
LOPE2	32.95 ± 1.47 ^d^	27.62 ± 1.18 ^b,c^	27.56 ± 1.24 ^c^	8.929 ± 0.319 ^c^	20.408 ± 0.833 ^c^
COPE1	75.12 ± 3.38 ^a^	26.51 ± 1.14 ^c^	42.47 ± 1.94 ^a^	9.901 ± 0.392 ^b^	32.258 ± 0.790 ^a^
COPE2	38.33 ± 1.53 ^c^	43.12 ± 1.74 ^a^	25.24 ± 1.12 ^c^	5.208 ± 0.216 ^d^	17.857 ± 0.318 ^d^

LOPE: *Leccino* cv Olive Pomace Extract; COPE: *Carolea* cv Olive Pomace Extract; (1) water/ethanol; (2) water/acetone. ARP = Antiradical Power; GAE = Gallic Acid Equivalent; CTE = (+)-Catechin Equivalent; TPC = Total Phenol Content; PAC = Phenolic Acid Content; FC = Flavonoid Content; DPPH = 2,2′-diphenyl-1-picrylhydrazyl radical; ABTS = 2,2′-azino-bis(3-ethylbenzothiazoline-6-sulphonic acid) radical. Different letters are significantly different at *p* < 0.05.

**Table 5:** Antioxidant characterization of starch conjugate polymers and corresponding leaving powders. Results are expressed as mean ± S.D. (*n* = 3), *p* < 0.05.

Code	TPC (mg GAE/g)	PAC (mg GAE/g)	FC (mg CTE/g)	ARP (mL mg^−1^)	
				DPPH Radical	ABTS Radical
SSE	49.94 ± 2.33 ^a^	19.46 ± 0.85 ^a^	14.56 ± 0.67 ^a^	4.762 ± 0.202 ^a^	5.556 ± 0.199 ^a^
BS	-	-	-	-	-
CS	-	-	-	-	-
LP-SSE	17.98 ± 0.74 ^b^	8.83 ± 0.31 ^b^	8.70 ± 0.34 ^b^	1.786 ± 0.054 ^b^	3.175 ± 0.142 ^b^
LP-BS	-	-	-	-	-
LP-CS	-	-	-	-	-

SSE: Starch conjugate with *Carolea* cv Olive Pomace Extracted in water ethanol; BS: Blank *starch polymer*; CS = Commercial starch; LP-SSE = Leaving powder from SSE; LP-BS = Leaving powder from BS; LP-CS = Leaving powder from CS. GAE = Gallic Acid Equivalent; CTE = (+)-Catechin Equivalent; ARP = Antiradical Power; TPC = Total Phenol Content; PAC = Phenolic Acid Content; FC = Flavonoid Content; DPPH = 2,2′-diphenyl-1-picrylhydrazyl radical; ABTS = 2,2′-azino-bis(3-ethylbenzothiazoline-6-sulphonic acid) radical. Different letters are significantly different at *p* < 0.05. (-) = not detected or below the limit of quantification.

**Table 6:** Antioxidant properties of the cookie extracts at different times. Results are expressed as mean ± S.D. (*n* = 3), *p* < 0.05.

Time (Days)	Code	TPC (mg GAE/g)	PAC (mg GAE/g)	FC (mg CTE/g)	ARP (mL mg^−1^)	
					DPPH Radical	ABTS Radical
	B-SSE	2.162 ± 0.078 ^a^	0.372 ± 0.015 ^a^	0.360 ± 0.014 ^a^	0.132 ± 0.005 ^a^	0.554 ± 0.010 ^b^
0	B-BS	0.551 ± 0.008 ^g^	-	-	-	-
	B-CS	0.771 ± 0.018 ^d^	-	-	-	-
2	B-SSE	1.992 ± 0.072 ^b^	0.352 ± 0.011 ^a,b^	0.342 ± 0.010 ^a^	0.129 ± 0.004 ^a^	0.512 ± 0.019 ^a,b^
	B-BS	0.533 ± 0.011 ^g,h^	-	-	-	-
	B-CS	0.742 ± 0.015 ^d,e^	-	-	-	-
4	B-SSE	1.975 ± 0.069 ^b,c^	0.332 ± 0.012 ^b,c^	0.311 ± 0.007 ^b^	0.124 ± 0.004^4^	0.497 ± 0.018 ^a^
	B-BS	0.522 ± 0.020 ^h,i^	-	-	-	-
	B-CS	0.723 ± 0.012 ^e^	-	-	-	-
6	B-SSE	1.964 ± 0.062 ^c^	0.315 ± 0.009 ^c^	0.294 ± 0.009 ^c^	0.123 ± 0.004 ^a^	0.478 ± 0.019 ^a^
	B-BS	0.493 ± 0.011 ^i^	-	-	-	-
	B-CS	0.692 ± 0.010 ^f^	-	-	-	-

B-SSE: Cookie based on LP-SSE; B-BS: Cookie based on LP-BS; B-CS = Cookie based on LP-CS. GAE = Gallic Acid Equivalent; CTE = (+)-Catechin Equivalent; ARP = Antiradical Power; TPC = Total Phenol Content; PAC = Phenolic Acid Content; FC = Flavonoid Content; DPPH = 2,2′-diphenyl-1-picrylhydrazyl radical; ABTS = 2,2′-azino-bis(3-ethylbenzothiazoline-6-sulphonic acid) radical. Different letters are significantly different at *p* < 0.05. (-) = not detected or below the limit of quantification.

**Table 7:** Oxitest results, expressed as induction period (hours, h).

Sample	Induction Period (IP, h)
B-CS	26 ± 2 ^a^
B-SSE	37 ± 2 ^b^
sign.	***

B-SSE: Cookie based on LP-SSE; B-CS: Cookie based on LP-CS. Legend: data followed by different superscript letters, in the same column, are significantly different (significant difference *t*-test, *p* < 0.05); asterisks indicate significance at *** *p* < 0.001.

**Table 8:** Moisture, water activity, and texture characteristics in cookies packaged and stored in ASL condition (40 °C, 55% R.H.).

Time (Day)		0	7	14	21	42	sign.
Moisture (%)	B-CS	3.71 ± 0.12 ^bB^	3.59 ± 0.17 ^bB^	2.88 ± 0.25 ^aB^	2.63 ± 0.42 ^a^	2.39 ± 0.57 ^a^	**
	B-SSE	3.27 ± 0.15 ^dA^	2.53 ± 0.10 ^cA^	2.47 ± 0.01 ^cA^	2.29 ± 0.08 ^b^	2.12 ± 0.19 ^a^	**
	sign.	***	***	***	n.s.	n.s.	
Water activity	B-CS	0.430 ± 0.026 ^b^	0.410 ± 0.007 ^b^	0.429 ± 0.006 ^b^	0.346 ± 0.006 ^a^	0.316 ± 0.028 ^a^	***
	B-SSE	0.420 ± 0.014 ^b^	0.412 ± 0.019 ^b^	0.410 ± 0.017 ^b^	0.361 ± 0.025 ^a^	0.333 ± 0.026 ^a^	***
	sign.	n.s.	n.s.	n.s.	n.s.	n.s.	
Deformability (mm)	B-CS	2.53 ± 1.26 ^a^	2.65 ± 1.15 ^a^	3.13 ± 0.12 ^a^	3.68 ± 0.37 ^b^	3.80 ± 0.17 ^b^	**
	B-SSE	1.72 ± 1.06	2.37 ± 1.16	2.12 ± 1.06	2.79 ± 1.03	2.77 ± 0.8	n.s.
	sign.	n.s.	n.s.	n.s.	n.s.	n.s.	
Friability (mm^−1^)	B-CS	0.50 ± 0.25 ^ab^	0.47 ± 0.25 ^ab^	0.32 ± 0.01 ^a^	0.27 ± 0.03 ^a^	0.77 ± 0.17 ^b^	**
	B-SSE	0.73 ± 0.28	0.55 ± 0.31	0.56 ± 0.21	0.44 ± 0.26	0.58 ± 0.19	n.s.
	sign.	n.s.	n.s.	n.s.	n.s.	n.s.	

B-SSE: Cookie based on LP-SSE; B-CS: Cookie based on LP-CS. Data followed by different superscript capital letters, in the same column, resulted significantly different to *t*-test (*p* < 0.05); data followed by different superscript lowercase letters, in the same line, resulted significantly different to Tukey-test (*p* < 0.05); asterisks indicate significance at: ** *p* < 0.01; *** *p* < 0.001, n.s. not significant.

**Table 9:** Color in cookies packaged and stored in ASL condition (40 °C, 55% R.H.).

Time (Day)		0	7	14	21	42	sign.
L*	B-CS	80.09 ± 2.07 ^aB^	81.75 ± 1.45 ^bB^	81.40 ± 1.50 ^bB^	82.11 ± 1.38 ^bB^	81.41 ± 1.39 ^bB^	*
	B-SSE	65.09 ± 0.39 ^A^	65.68 ± 0.69 ^A^	65.10 ± 0.44 ^A^	66.06 ± 0.54 ^A^	65.30 ± 0.39 ^A^	n.s.
	sign.	***	***	***	***	***	
a*	B-CS	−0.46 ± 0.03 ^cA^	−0.78 ± 0.05 ^aA^	−0.44 ± 0.02 ^cA^	−0.64 ± 0.03 ^bA^	0.04 ± 0.07 ^dA^	*
	B-SSE	5.12 ± 0.08 ^aB^	5.22 ± 0.08 ^aB^	5.76 ± 0.07 ^bB^	5.01 ± 0.13 ^aB^	5.64 ± 0.26 ^bB^	*
	sign.	***	***	***	***	***	
b*	B-CS	28.11 ± 0.96 ^bB^	27.61 ± 1.43 ^bB^	28.08 ± 0.59 ^bB^	27.13 ± 0.62 ^aB^	27.00 ± 0.63 ^aB^	*
	B-SSE	26.35 ± 0.18 ^A^	26.59 ± 0.19 ^A^	26.84 ± 0.08 ^A^	26.18 ± 0.35 ^A^	26.38 ± 0.22 ^A^	n.s.
	sign.	*	*	*	*	*	
C	B-CS	28.15 ± 0.90 ^b^	27.68 ± 1.48 ^abB^	28.13 ± 0.61 ^b^	27.17 ± 0.67 ^aB^	27.06 ± 0.54 ^a^	*
	B-SSE	26.85 ± 0.38 ^ab^	26.27 ± 0.08 ^aA^	27.43 ± 0.30 ^bB^	26.66 ± 0.26 ^abA^	26.98 ± 0.25 ^ab^	*
	sign.	n.s.	*	n.s.	*	n.s.	
h°	B-CS	91.04 ± 2.00 ^abB^	91.97 ± 1.54 ^bB^	91.10 ± 1.08 ^abB^	91.68 ± 0.83 ^abB^	90.32 ± 1.15 ^aB^	*
	B-SSE	78.96 ± 0.30	78.92 ± 0.38	77.95 ± 0.39	79.19 ± 0.28	77.96 ± 0.21	n.s.
	sign.	***	***	***	***	***	

B-SSE: Cookie based on LP-SSE; B-CS: Cookie based on LP-CS. Data followed by different superscript capital letters, in the same column, resulted significantly different to *t*-test (*p* < 0.05); data followed by different superscript lowercase letters, in the same line, resulted significantly different to Tukey-test (*p* < 0.05); asterisks indicate significance at: * *p* < 0.05; *** *p* < 0.001, n.s. not significant.

**Table 10:** Total color differences (ΔE) are calculated for: (a) B-CS and B-SSE during the ASL (T*_n_*, time of observation; T_0_, initial time); (b) two samples at the same time of observation (B-SSE vs. B-CS).

	T_n_ vs. T_0_		B-SSE vs. B-CS
*n*	B-CS	B-SSE	
0	--	--	16.1
7	1.8	0.6	17.2
14	1.6	0.6	17.4
21	2.6	1.1	17.3
42	1.9	1.0	17.2

## Data Availability

The original contributions presented in this study are included in the article/Supplementary Materials. Further inquiries can be directed to the corresponding author.

## Supplementary Materials

The following supporting information can be downloaded at: https://www.mdpi.com/article/10.3390/foods15091488/s1. S1. Total phenolic content determination; S2. Total phenolic acid determination; S3. Flavonoid content determination; S4. Scavenger activity against DPPH radical; S5. Scavenger activity against ABTS radical; Figure S1. Oxidation evaluation measured by the Oxitest oxidation stability reactor. Estimation of the induction period of the sample (after the cooking step) in contact with oxygen (0.6 MPa).
