# Supplementary material for: Valorization of Olive Milling By-Products: Development and Application of an Antioxidant-Enriched Leavening Powder for Bakery Products
Source: Foods. 2026 Apr 24;15(9):1488. doi: 10.3390/foods15091488 (PMC13164460; doi:10.3390/foods15091488)
Supplement: Supplementary file 1 [file foods-15-01488-s001.zip › foods-4237535-supplementary.pdf]

SUPPORTING INFORMATION

Valorization of Olive Milling By-Products: Development and Application of an Antioxidant-Enriched Leavening Powder for Bakery Products

Table of Contents

S1. Disposable phenolic groups by the Folin–Ciocalteu procedure

S2. Total phenolic acid determination

S3. Flavonoid content determination

S4. Scavenger activity against DPPH radical

S5. Scavenger activity against ABTS radical

S1. Total phenolic content determination

For each sample, an aqueous solution at different concentrations of the sample was prepared, and 1 mL of Folin–Ciocalteu reagent was added to 6 mL of solution, and, after 3 minutes, 3 mL of aqueous

solution of Na_2CO_3 (2% w/v). The solutions thus prepared were kept under stirring in the dark for 2 h. The absorbance of the samples was then spectrophotometrically measured at the wavelength of 760 nm (Evolution 201 spectrophotometer (Thermo Fisher Scientific, Hillsboro, OR, USA)), against a control solution prepared under the same conditions, but containing 6 mL of purified water. The amount of available phenolic groups was expressed as milligrams of gallic acid (GA) equivalent *per* gram of sample (mg GAE/g sample). A calibration curve was constructed, using aqueous solutions of GA at different concentrations (8.0, 16.0, 24.0, 32.0, and 40.0 μM). To define the regression line and the R^2 coefficient using the least squares method, the concentrations of the aqueous solutions of GA were correlated to the respective absorbance values.

S2. Total phenolic acid determination

At 1 mL of an aqueous solutions of each sample were added 5.0 mL of purified water, 1.0 mL of HCl 0.5 mol L^{-1}, 1.0 mL of the Arnov's reagent (10.0 g of sodium molybdate and 10.0 g of sodium nitrite were solubilized in 100 mL of purified water) and 1.0 mL of NaOH (4.0% w/v), obtaining a final volume of 10 mL with purified water. A control solution was also prepared under the same conditions by replacing the sample solution with purified water. The absorbance of the samples was measured spectrophotometrically at 490 nm (Evolution 201 spectrophotometer (Thermo Fisher Scientific, Hillsboro, OR, USA).

S3. Flavonoid content determination

Aqueous solutions of each sample were prepared at different concentrations, and to 0.5 mL of each, 2.0 mL of purified water and 0.15 mL of $NaNO_2$ aqueous solution (15% w/v) were added. After 6 minutes, 0.15 mL of $AlCl_3$ solution (10% w/v) was added to the stock solution. Subsequently, after 6 minutes, 3.0 mL of NaOH solution (4% w/v) was added, obtaining a final volume of 5 mL with purified water. The solution thus prepared was kept away from light for 15 minutes. A control solution was also prepared under the same conditions, but without a sample. The absorbance of the solutions

was then measured with a spectrophotometer at a wavelength of 510 nm (Evolution 201 spectrophotometer (Thermo Fisher Scientific, Hillsboro, OR, USA). The total content of flavonoids was expressed in milligrams of catechin per gram of sample (mg CT / g sample), after carrying out a suitable calibration curve.

S4. Scavenger activity against DPPH radical

Aqueous solutions of each sample were prepared at different concentrations, and, at 1.0 mL every solution 4.0 mL of purified water and 5.0 mL of an ethanolic solution of DPPH (200 µmol L^{-1}) were added, obtaining a radical concentration of 100 µmol L^{-1}. The mixture was kept at 25°C for 30 minutes, and the residual concentration of the DPPH radical was evaluated with a spectrophotometer at 517 nm. The percentage of inhibition of the DPPH radical species was calculated according to the following formula:

$$\text{Inhibition (\%)} = (A_0 - A_1)/A_0 \times 100$$

where A_0 is the absorbance of the control prepared under the same conditions using 5 mL of purified water, and A_1 is the absorbance of each examined sample. The scavenging activity on the lipophilic DPPH radical was expressed in terms of IC_{50}.

S5. Scavenger activity against ABTS radical

The assay that allows for evaluating the antioxidant capacity of the different samples exploits the scavenging activity of a given compound against the ABTS radical. Different volumes of the aqueous solutions of each sample were added to 2 mL of ABTS radical solution. The solutions were kept in the dark for 6 minutes, and the residual concentration of the ABTS radical was spectrophotometrically evaluated at 734 nm. The percentage of inhibition of the ABTS radical was calculated according to the following formula:

$$\text{Inhibition (\%)} = (A_0 - A_1)/A_0 \times 100$$

where A_0 is the absorbance of the control prepared under the same conditions using 0.5 mL of purified water, and A_1 is the absorbance of each examined sample. The scavenging activity of the analyzed system was expressed in terms of IC_{50}.

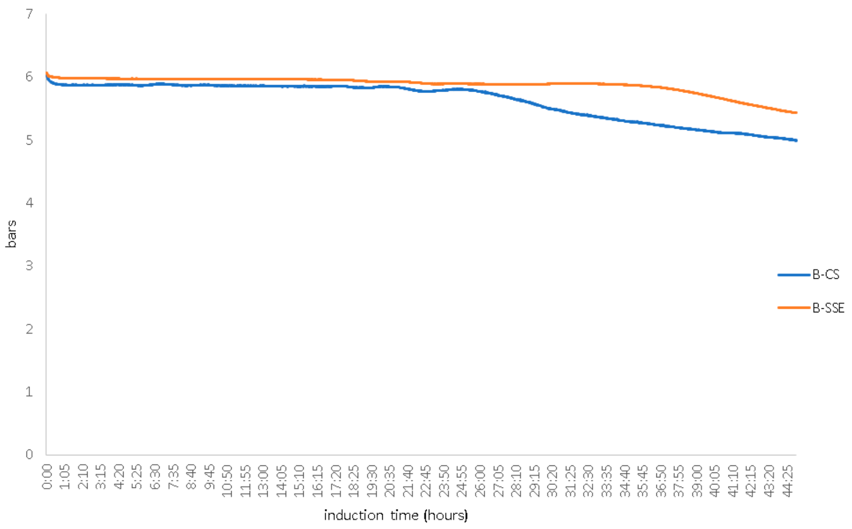

Figure S1. Oxidation evaluation measured by the Oxitest oxidation stability reactor. Estimation of the induction period of the sample (after the cooking step) in contact with oxygen (0.6 MPa).
